# “I think they should give primary health care a little more priority”. The primary health care in Caribbean SIDS: what can be said about adaptation to the changing climate? The case of Dominica— a qualitative study

**DOI:** 10.1186/s12875-024-02311-w

**Published:** 2024-02-22

**Authors:** Fiona Harris-Glenville, Patrick Cloos

**Affiliations:** 1 Faculty of Medical Sciences, Department of Community Health and Psychiatry, The University of the West Indies (Mona campus), Kingston, Jamaica; 2 School of Social Work and Department of Social and Preventive Medicine (School of Public Health); Centre de Recherche en Santé Publique, University of Montreal, Montréal, Canada

**Keywords:** Climate change, Primary health care, Caribbean, West Indies, Small islands developing states, Adaptation, Vulnerability, Essential public health functions, Essential environmental public health functions

## Abstract

**Background:**

Adaptation to climate change (CC) is a priority for Small Island Developing States (SIDS) in the Caribbean, as these countries and territories are particularly vulnerable to climate-related events. Primary health care (PHC) is an important contributor to CC adaptation. However, knowledge on how PHC is prepared for CC in Caribbean SIDS is very limited. The aim of this paper is to discuss health system adaptation to climate change, with a focus on PHC.

**Methods:**

We explored the perspectives of PHC professionals in Dominica on PHC adaptation to climate change. Focus group discussions (FGDs) were conducted in each of the seven health districts in Dominica, a Caribbean SIDS, between November 2021 and January 2022. The semi-structured interview guide was based on the Essential Public Health Functions: assessment, access to health care services, policy development and resource allocation. Data coding was organized accordingly.

**Results:**

Findings suggest that health care providers perceive climate change as contributing to an increase in NCDs and mental health problems. Climate-related events create barriers to care and exacerbate the chronic deficiencies within the health system, especially in the absence of high-level policy support. Healthcare providers need to take a holistic view of health and act accordingly in terms of disease prevention and health promotion, epidemiological surveillance, and ensuring the widest possible access to healthcare, with a particular focus on the environmental and social determinants of vulnerability.

**Conclusion:**

The primary health care system is a key stakeholder in the design and operationalization of adaptation and transformative resilience. The Essential Public Health Functions should integrate social and climate and other environmental determinants of health to guide primary care activities to protect the health of communities. This study highlights the need for improved research on the linkages between climate events and health outcomes, surveillance, and development of plans informed by contextual knowledge in the SIDS.

**Supplementary Information:**

The online version contains supplementary material available at 10.1186/s12875-024-02311-w.

## Introduction

Anthropogenic climate change and the associated global warming are due to the emission of greenhouse gases, particularly carbon dioxide, occurring as consequences of exploitation and combustion of fossil fuels for energy and industrial purposes [1]. Climate change (CC) is a global public health issue with serious consequences for biodiversity, human health, and societies [2]. Climate extremes (extreme weather or climate events such as hurricanes, storms, heat waves, extreme precipitation and flooding, droughts) and slow-onset events (such as sea level rise ocean acidification, temperature increases, desertification, land degradation, loss of biodiversity) interact with the social determinants of health (poverty, housing, employment, human mobility, conflict, access to health care services) to influence human health outcomes (malnutrition, mental health, chronic diseases, infectious and vector-borne diseases) [3]. Exposure and vulnerability to climate change and its associated risks are also determined by social and political factors, and more generally the development context, which can trigger human mobility and influence health outcomes [4]. Climate change can be seen as an issue of (in-)justice and (in-)equity in the sense that it generates health inequalities due to differences in exposure, vulnerability and coping capacity between and within countries [5]. Preparation of societies to respond to the adverse effects of climate change on public health is therefore urgent and adaptation measures must be taken and implemented to reduce vulnerabilities [1, 6].

In the Sixth Assessment Report of the United Nations (UN) Intergovernmental Panel on Climate Change (IPCC 6), adaptation refers to the process of adjusting to actual or expected climate and its impacts, in order to mitigate harm or exploit beneficial activities [1]. Adaptation measures include raising public awareness, reducing vulnerability to extreme climate events, spatial planning and land use, identifying risks and exposures, establishing early warning systems, and protecting human health [1].

### Health adaptation in Caribbean SIDS: focus on primary health care services

The Caribbean has been described as particularly vulnerable to climate extremes such as tropical storms or hurricanes [7–10] which can trigger human mobility (internal displacement and international migration) [11]. The health sector including health care services and health professionals can play a leading role in contributing to the adaptation to CC to protect the health of the population [12–14].

In the Caribbean, there has been some efforts by regional health agencies to support health system adaptation and resilience to CC. Strengthening the capacity of the health sector to adapt to climate change, and other environmental determinants of health, while focusing on conditions of vulnerability and the social determinants of health, is recognized as a priority by the Pan American Health Organization (PAHO) to protect the health of the populations [15]. In 2019, PAHO/WHO, in consultation with the Ministries of health of Caribbean SIDS, developed a Caribbean Action Plan on Climate Change and Health. This initiative recognizes the importance of including health at the center of all plans for climate change adaptation and preparedness. It proposes guiding strategies for Caribbean SIDS to support health leadership; develop research, surveillance and policies on health impacts and CC (including vulnerability and adaptation assessments); and prepare health systems for climate risks [8].

Greater involvement of the health care system and public health actors, in the response and preparedness for climate extremes in Caribbean SIDS has been recommended [16]. More specifically, the strengthening of primary health care services is a public health initiative that can support adaptation to climate extremes to protect the health of the population, by supporting communities, especially those considered most vulnerable to climate extremes [17]. The primary health care model of service delivery is widespread in the Caribbean, as countries in the region have adopted the WHO recommendations emerging from the Alma Ata Declaration in 1978, with the aim of promoting universal access to health care services [18, 19].

There is a paucity of research and knowledge in Caribbean SIDS on climate change and health, and on the overall level of public health preparedness for climate-related events [20]. Considering this, the overall objective of this research is to better understand how the primary health care (PHC) system in Caribbean SIDS is adapting to the changing climate. It seeks to explore the perspectives of professionals providing primary health care services, on climate change and its social and health impacts, and their perceptions of what the primary health care system could do to contribute to climate change adaptation in Caribbean SIDS. The knowledge gained from this study can be used to support climate change adaptation efforts in the Caribbean.

### Theoretical framework

Our study is guided by the Essential Public Health Functions (EPHF) framework, which was recently adapted by PAHO into the Essential Environmental Public Health Functions (EEPHF) framework. These public health functions represent a theoretical and integrated model that includes four interrelated components – health assessment, access, resources allocation and policy development – to assist the public health sector to address the environmental determinants of health, including climate change, as well as political, social and cultural factors that contribute to health inequities [21]. EPHF is considered as a key component in achieving universal health coverage (UHC) [22] and the health-related targets of the Sustainable Development Goals (SDGs) [23].

**Fig. 1:**
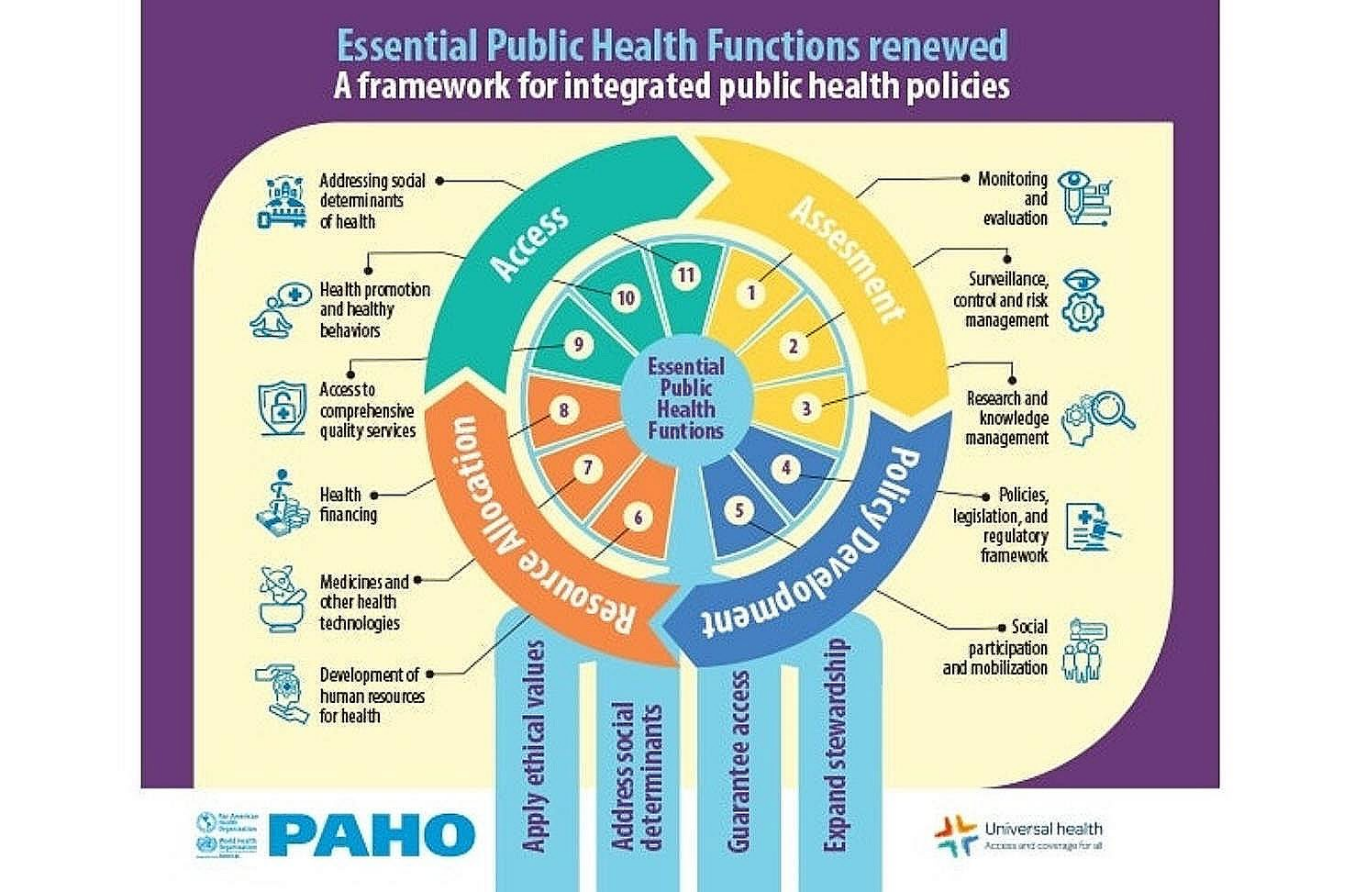
PAHO Essential Public Health Functions Framework Source: *The Essential Environmental Public Health Functions. A framework to implement the Agenda for the Americas on Health, Environment, and Climate Change 2021–2030*

## Methods

### Study context: the case of Dominica, a caribbean SIDS

This qualitative study is part of a larger research project conducted by PC and colleagues that is entitled «Climate, Migration and Health in the Caribbean (*CliMiHealth*)». The aim of this interdisciplinary study is to better understand how climate change affects human mobility and the health of populations in Caribbean SIDS, particularly in Dominica. PC, as a physician with a public health background, contributed to a health assessment in Dominica with *Médecins sans Frontières* (MSF) in September 2017, immediately following the impact of Hurricane Maria in Dominica. PC also served as the Chief Medical Officer in Dominica from 2003 to 2005 and supervised the PHC system. FHG works as a nurse in the PHC system and is pursuing a doctoral degree in public health. This reflects the interest that the authors of this article have in Dominica and the Caribbean, and in the domain of public health which is a multidisciplinary field of knowledge and action to improve or protect the health of the population.

Dominica is a mountainous island located in the Eastern Caribbean, the largest of the Windward islands with an estimated population of 70,000. It is classified as an upper middle-income country with GNI per capita income of US$ 7790 in 2021. The country faces social challenges of poverty, unemployment, and inequality in the distribution of wealth and resources. The 2009 country poverty assessment found that 29% of the population lived below the poverty line and youth unemployment was at 31%. The GINI ratio for that year was at 0.44 [24]. Approximately 4% of the Dominican population is classified as Kalinago (indigenous population) and this population, often referred to as vulnerable, faces historical and current environmental, social and health challenges [25].

Life expectancy at birth was estimated to be 78 years (2021). Public expenditure on health was about 3.5% of GDP in 2019, comparable to Trinidad and Tobago (3.2%) and Jamaica (4%) [26]. Hypertension and diabetes are the most common noncommunicable diseases (NCDs) treated in primary care clinics in Dominica, accounting for up to 90% of clinic visits [27]. Chronic diseases were already a major problem in Dominica in the 1990s [20] and remain so today [26].

Since the early 1980’s health services in Dominica have been organized according to the principles of primary health care services: universal access, integration and decentralization [27]. The health system is centrally managed and consists of primary and secondary health care services. Primary health care services are provided through seven health districts, grouped into two regions. Each health region is supervised by a senior community health nurse (SCHN) who supports the national PHC Director [28]. Morbidity data are collected at the district (primary care) and the hospital (secondary care) levels and sent to the Health Information Unit. The quality of some health care services in Dominica has recently been questioned in the context of a dramatic increase in neonatal mortality between 2000 and 2016 (mostly due to intrapartum complications) [29].

Several climate-sensitive health risks are of concerns for Dominica such as water and food insecurity, vector-borne diseases, mental health issues, and non-communicable diseases [30]. Dominica has a long history of climate extremes particularly storms and hurricanes [24, 31]. Dominica has an Office of Disaster Management whose role is to inform the public about multiple hazards (e.g., volcanoes, hurricanes, flooding, landslides), and organize responses and minimize their impacts (odm.gov.dm). Two recent events highlighted the need to better prepare for and adapt to climate change. In 2015, the country was hit by Tropical Storm Erica which caused more than EC$1.3 billion (US$483 million) in damage and losses, equivalent to approximately 90% of Dominica’s Gross Domestic Product (GDP). It resulted in loss of life and property, population displacement and loss of livelihood for many Dominicans [32]. In 2017, the country was again devastated by Hurricane Maria, which caused severe damage and loss. The health sector was severely impacted, with more than 80% of the infrastructure damaged and the need for building greater resilience within the health system was identified [33].

Following the impact of Hurricane Maria, the Government of Dominica established the Climate Resilience Execution Agency (CREAD), with the funding from the Government of Canada, to support reconstruction and recovery projects and develop a plan for ensuring the countries’ resilience to climate change. However, the CREAD 2020–2030 plan makes limited reference to the health system and no reference to the involvement of PHC in this climate resilience process [34].

### Study design

To better understand the contribution of the primary health care system in Dominica in the process of adapting to a changing climate, we conducted a qualitative study to explore the perspectives of primary health care professionals in Dominica.

### Sampling, recruitment, and data collection

Several meetings were held with representatives of the Ministry of Health in Dominica to present and discuss the *CliMiHealth* study and its objectives, and the plan to conduct focus group discussions (FGDs) with PHC staff. Community Health Nurses (CHN) were also contacted by telephone to discuss the organization of FGDs. Inclusion criteria was to be a professional working in the PHC system in Dominica at the time of the study. The sampling strategy was guided by diversification based on health districts and PHC professional categories. A poster was designed and shared with the CHNs to inform and invite PHC professionals and assistive personnel to participate in the FGDs. A soft copy of the consent form was also shared to provide more details about the study. Seven FGDs (one in each of the seven health districts) were conducted between November 2021 and January 2022 at the date and time agreed on with the CHNs.

Inspired by the EPHF framework, a semi-structured focus-group guide (Annex A) was constructed collaboratively around the following themes: assessment, policy development, resource allocation and access to health care services. The seven FGDs were organized and conducted by FHG, with the contribution of PC and NMP, in a room at the main health center in each district. Each FGD lasted between 40 and 60 min and consisted of 5 to 12 participants. Participants were reminded of their right to refuse or opt-out of the study prior to FGD commencement.

A one-day «Primary Health Care and Climate Change Research Workshop» was held in Dominica in July 2022. After meetings and discussions with the PHC Director and Senior Community Health Nurses, it was agreed that they would contact the PHC teams to participate in the workshop. It was finally organized and presented by PC in collaboration with FHG to a group made of 27 participants from the seven health districts. The objectives of the workshop were to discuss and validate the main findings of the FGDs, and to use the opportunity to raise awareness on climate change and health. Each presentation was made by PC with the collaboration of FHG followed by small group and plenary discussions on three main themes: impacts of CC on living conditions, public health and health care services; health assessment (including risks and vulnerabilities); and preparation/adaptation (including needs of training, education, resource and support) of the PHC system to climate change. Each thematic discussion lasted approximately one hour. Presentations and plenary sessions were recorded, and notes were taken by 2 observers.

### Data analysis

All focus group sessions were transcribed verbatim by FHG. The transcriptions were uploaded into QDA Miner© for data processing and coding. Qualitative analysis was conducted by FHG and PC, and was inspired by Miles et al. (2019) [35]. Deductive coding using a pre-existing set of codes based on the framework and the interview guide was assigned to the data. Inductive coding allowed for other codes to emerge incrementally during data collection and analysis (e.g., knowledge about climate change and related events, communication, retroaction of information, increased need for health care, biomedical approach). Care was taken not to distort the meaning of the words. Notes from the workshop were compared with the transcripts to identify new or different information. In general, there was consensus between the information collected from the FGDs and the workshop and they were reported simultaneously.

## Results

### Characteristics of the focus groups

A total of 59 individuals participated in the seven FGDs (see Table 1). The majority (89.9%) of the participants were female and ranged in age from 27 to 62 years, with a mean age of 40.34 +/- 8.9 years. Nurses and Community Health Aides were the largest group represented (see Table 2).

**Table 1:** FGD location (Health Districts)

Location	Number of participants	Percentage
Castle Bruce	10	16.9
Grand Bay	7	11.9
La Plaine	7	11.9
Marigot	10	16.9
Portsmouth	5	8.5
Roseau	12	20.3
St Joseph	8	13.6

**Table 2:** Demographic information for participant of FGDs

	Number	Percentage
**Sex**		
Female	53	89.9
Male	6	10.1
**Level of Education**		
Graduate university diploma	11	18.6
Undergraduate University Diploma	14	23.7
Non-university post secondary diploma	23	39.0
Secondary School	9	15.3
Primary	2	3.4
**Category of Staff**		
Cleaner	1	1.7
Community Health Aide	13	22.0
Community Health Nurse	3	5.1
Driver	1	1.7
Environmental Health Officer	4	6.8
Family Nurse Practitioner	2	3.4
Medical Doctor	5	8.5
Pharmacist	3	5.1
Primary care nurse	1	1.7
Registered Nurse	21	35.6
Staff Nurse Midwife	5	8.5

*N = 59

### Findings

The four themes presented below are organized around the four categories and subcategories of the Essential Public Health Functions (Fig. 1). It is important to note that, in theory, these areas of activity are interrelated. However, this is not always the case in practice. We have included in each theme, the challenges that were expressed by the PHC staff in relation to each function (e.g. challenges related to health assessment).

### Health assessment: knowledge, monitoring and surveillance

In Dominica’s PHC, data collection on routine visits is ostensibly done manually on morbidity sheets or other ledgers at each point of care in the health districts. Medical records are in the form of notebooks kept by the patient. Data collection is fragmented with each program area having its own data collection system. Quarterly reports are sent to the Health Information Unit which operates at the central level. The following section of the manuscript focuses on the participants’ knowledge and observations related to climate change and health. This provides a general idea of the level of awareness of CC and health in the PHC system, and subsequently the capacity of professionals to adapt, prepare and respond to climate extremes. In terms of knowledge about climate change, some participants shared their observations about the local impact of climate change and how they think it relates to the health of the population. A few participants linked climate change to global warming, but the most common themes were ‘climate disasters’ and changes in seasonality. In general, the discussion focused on hurricanes and floods. Issues such as droughts, heat waves and sea level rise were rarely discussed, and it was clear that these aspects of climate change were underestimated. The following categories emerged from the discussions and are the result of the participants’ perspectives.

#### A change in seasonality

Dominica’s climate is considered to have two seasons – the rainy season which runs from June to the end of November and the dry season (or “Kawem” in Creole), which runs from early December to the end of May. Participants observed that this pattern has changed, and that this distinction is no longer apparent. One participant noted “*Now we no longer have a “kawem” or dry season and rainy season. It’s like we don’t see that pattern no more”.* Another stated: “*I think it’s more in a change in the weather pattern … During the rainy season it was dry and during the dry season you had a lot of rain” (FGD7).* This change in seasonality was noted to have an impact on the availability of local agricultural produce and therefore on people’s diets.

#### Intensification of extreme weather events: hurricanes, heat, rainfall, floods, landslides

Some participants explained that there has been an increase in the intensity of hurricanes in Dominica: “*well the hurricanes have intensified. Look at Maria, that was the worst we have ever experienced.”* (FGD7). Regarding heat waves: *“yes, we’ve been having some serious uprise in heat.” (FGD 4)*. According to the participants, the frequency of floods and landslides has increased, especially in areas that are not usually flooded “*I think we are having more frequent floods. Because when I was younger you would never think of Dominica having floods.” (FGD2)*. Respondents explained that even with very little rainfall, flooding and landslides occur, causing extensive damage to property and disrupting of livelihoods. “*Because we have never seen in our lifetime our river overflowing its bank so heavily and that caused a lot of the houses [to be damaged] the buildings were torn apart, washed away. Villagers were relocated, some had to rebuild” (FGD1).* In addition, some participants reported that there were tropical storms outside of the normal hurricane season including during the month of December which was considered unexpected. In at least 4 of the FGDs, participants indicated an increased frequency and intensity of rainfall during the Christmas season which seemed abnormal: “*take December for example, for the past Christmases, we have had tropical storm weather on Christmas days! Usually we might have light drizzle during the Christmas season but not that magnitude where you can barely walk through Roseau and you have flooding”* (FGD4); *“we have rain so often in this month of December which is not normal.” (FGD6)*.

#### Warming temperatures

One participant described the warming of the atmosphere over the last 10 years: “*10 years ago it didn’t really used to be so hot and as the years go by it’s getting hotter and hotter every day” (FGD2)*. It was noted that the increase in heat has led to a reduction in physical activity among people accessing the primary healthcare system. In contrast, in some FGDs, particularly in the rural areas, heat waves were not seen as a problem at all “*Our heat waves, here in Dominica we do not have heat waves like other countries where… We do get it but it’s not that bad…” (FGD3).* It was also not clear what the participants identified as heat waves.

#### Intensification of Saharan dust, sea weed, and black flies

Some participants mentioned Saharan dust, indicating that there was a perceived increase in the frequency and quantity of Saharan dust and that this was perceived to be linked to climate change. Others mentioned the increase in seaweed washing ashore, particularly in the Atlantic Ocean and the appearance of black flies along the coastal areas as being related to climate change.

### Health issues associated with climate change

Several health issues related to climate change were discussed: injuries, communicable and noncommunicable diseases, and mental health.

### Injuries and communicable diseases

Participants discussed health issues considered to be directly or indirectly related to climate change. Physical injuries and infections that can occur in the immediate aftermath of an extreme climate event such as a hurricane were reported as direct health problems related to climate change. These include health problems such as wounds and fractures, wound infections, gastroenteritis, dengue, and other vector-borne diseases. As a result, PHC staff are alert to these conditions, especially after hurricanes and storms.

#### Noncommunicable diseases

Participants linked climate change to an increase in the burden of NCDs, particularly hypertension and diabetes. *“I think all of us can attest that there has been a rise in new conditions post Hurricane Maria, especially high blood pressure and diabetes and stroke in younger persons, so maybe there is some relation, we did not conduct a survey or research to prove that but ….” (FGD6).* In other words, these observations from the field do not seem to be validated by systematic surveillance activities of chronic diseases, a situation that may impede systematic prevention and health promotion activities that should be conducted in primary care at the national level. In addition, it was suggested that there is a change in diet following extreme weather events such as storms, with increased consumption of processed foods. This change in diet was thought to be associated with an increase in new cases of hypertension and diabetes as well as affecting the blood pressure and/or glycemic control of patients who are already diagnosed. Some participants also suggested that climate change threatens food security particularly through the destruction of agricultural products due to climate related events.

#### Mental health issues

Mental health issues were also identified as an indirect effect of climate change. Participants perceived that mental health problems such as anxiety and depression are related to fear during events, loss of property and loved ones, loss of livelihoods and other social problems that may arise after an extreme climate event. Participants noted that mental health problems often go undiagnosed and untreated. For people with other NCDs, these mental health challenges sometimes contribute to non-adherence to treatment and lack of disease control. Participants reported that the social and physical challenges faced in the aftermath of extreme climatic events have psychological effects that last beyond the emergency period. “*some people develop mental disorders, psycho-social problems, they no longer can interact with others socially… mental illness is on a rise in our country… and there is no statistics necessary to show you the evidence of that because you can see it, and the younger people are becoming more mentally ill” (FGD 1).* Participants explained that the psychological effects of extreme climate events are long-lasting. People have become traumatized to the point where a small amount of rainfall causes extreme anxiety: “*what I have noticed is that people have become affected psychologically where based on their experience during the event of a disaster that they are unable to sleep at night they complain about being fearful, they are traumatized…” (FGD 4).* Staff also indicated that they too face mental health challenges. They face similar stressors as the rest of the population and they perceived that their needs are rarely addressed: “*I mean they (the PHC Staff) suffered. They suffered during the storms and hurricanes. Some of them were displaced, some of them lost family members or their property. So it impacted very heavily on them. Some of those health care providers were not able to provide healthcare services because they themselves were affected” (FGD3).*

#### Assessing climate risks and vulnerabilities

According to the participants, assessment of risks and vulnerabilities are activities that are carried out in the context of preparing for and responding to potential climate disasters. During the discussions regarding vulnerability, it was said that *‘our disaster plan is reviewed every year. Before the hurricane season at least we try to update it and to identify the vulnerable population and areas too and usually our disaster committee collaborates with us’ (FGD 2)*. In case of an event such as a storm or flood, people identified as ‘vulnerable’ or ‘at risk’ could be evacuated. However, it was unclear how the vulnerability assessment was carried out. There is a pre-existing list of ‘vulnerable populations’ based on age and medical conditions. Participants identified the elderly, older people living alone, pregnant women, children under 5, shut-in (home bound) and people with disabilities as vulnerable. Some mentioned of the housing status as an indicator of vulnerability and pointed out the lack of attention to social conditions that contribute to vulnerability. One participant said: *“sometimes we forget the social aspects of how people live” (FGD 2)*. In relation to land issues, the following participant said: *“I consider people vulnerable when they live in areas prone to landslides and floods” (FGD 2)*. Thus, vulnerability is also linked to the exposure to land conditions: *“we have some areas that are severely compromised. Every time it rains heavily and [there needs to be] river walls to prevent water from flowing into the community and into people’s homes. We are way in the center of the community, so it happens all the time. If we do not correct these situations it is going to happen all the time” (FGD 1).* Thus, land planning appears to be a concern, but it is unclear whether such an observation leads to action. Participants reported that there are manual records of community statistics that help to identify individuals who are considered to be at-risk. These records are segmented based on the program areas and used to create the community profile which is a snapshot of the community’s needs based on indicators such as age, medical conditions, and income status. It was unclear how often the community profile is updated and how it is used by the PHC for planning purposes.

Some were particularly concerned about the issue of internal displacement following a climate extreme. One participant lamented: “*We also see that the displacement of certain families, where they have to separate from each other due to accommodation, we see children and the elderly, vulnerable groups where they can be affected, where children may be exposed to abuse” (FGD3*). On the other hand, some participants felt that displaced populations were not a priority group because they would somehow find their way home: “*They make their way home, they pass by the sea. A lot of persons got trapped in places where they would not have been for Hurricane Maria and even TS Erica but they made their way home” (FGD 3).* Thus, many social conditions seem to be recognized as problematic, but it is not clear whether these observations lead to systematic health care interventions other than hurricane preparedness and response (e.g. evacuation of individuals or families considered vulnerable or at risk). Moreover, PHC activities do not appear to be guided by a systematic assessment of both climate risks and vulnerabilities.

#### Challenges with health assessment: a lack of feedback between departments in the health sector

Participants noted the inadequacy of the feedback mechanism for surveillance data. PHC staff record clinical information in general morbidity reports and this information is submitted to and analyzed by the Health Information Unit (HIU). This morbidity information relates to health problems such as chronic diseases (e.g. sickle cell anemia, asthma, diabetes, hypertension) and communicable diseases (genital or eye infections or unspecified), injuries, mental health conditions, or even common symptoms (e.g., fever, pain, headache). However, there appears to be limited systematic feedback from the HIU to the PHC staff. One participant state: *“ The only thing I want to put it on record is that we send the information up but we don’t get feedback. And the information is there… And somebody gets it but it never filters down…well report comes but we get it like everybody else, like general public, on the radio or on social media but it doesn’t come back to us.”* The lack of feedback calls into question the capacity of the disease surveillance system to inform public health interventions, decision-making and primary health care practices to protect or even improve the health of the population.

### Access to health care services

The issue of access to health care services is closely linked to the health assessment function, and involves the implementation of measures to ensure access to individual and population-based interventions.

#### Health care services driven by field observations and the interaction with the community

Staff seemed to respond to health issues based on their observations and not necessarily based on empirical data and systematic surveillance systems (e.g., nutritional status, mental health). Participants indicated that home and household visits are part of the routine services provided by the PHC and this allows for a close interaction with the community thereby affording the opportunity to adequately assess health needs: *“That’s the beauty of having district nurses because everybody knows everybody. So, the persons are aware of their nurses so they will find a way to pass the message to you. if they cannot call, they will send a message thru somebody so it will get to us”* (FGD 3). However, this calls into question both the level of assessment and access to primary health care and points to a lack of policy guidance. In some communities, staff conduct a walkthrough in the community in the immediate emergency period following extreme climate events. There are no guidelines for how often this is done or what the assessment entails. According to participants PHC teams in Dominica know the location of the people who have been identified as vulnerable: *“Because (it)is a small community. All the nurses know the(ir) people. So I know (that) if you(are) living in a block house, (with a ) concrete roof you may not need the primary initial care as opposed to someone who was in a wooden structure, who was displaced without treatment and without food” (FGD 3).* It appears that staff had different interpretations of what needed to be “known” about the community. The (in-) ability to assess health is therefore strongly linked to (lack of) access to care and support.

### Barriers to accessing health care

Some expressed concern about barriers to accessing health care such as when someone lives alone and is seriously ill or living with a disability. The following themes were related to barriers to care identified by participants.

#### Communication challenges in accessing health care services

Participants reported that communication challenges were a significant barrier to accessing to care. Some individuals and families are inaccessible due to their location and particularly in the context of climate extremes, communication challenges can prevent them from receiving the health care they need in a timely manner. When telecommunications services are down, individuals must rely on the goodwill of relatives and neighbors and their access to help is not always timely: “*If someone fall sick, they cannot access, they cannot call the ambulance or anyone for that matter to come and pick them up. So, they may have to rely on maybe a close neighbor or maybe their child or someone to run and call somebody else…” (FGD 2).* They noted that some people only receive needed health services if they are identified by the staff during home or household visits and these visits have limited frequency:

“*So, we have to just sit and hope that they show up. Sometimes we would go to the homes and the homes would be badly destroyed and we (are) not even sure that they moved to a neighbor or if they moved out of the district or out of state. We don’t know. So, it made that part of primary health care difficult.’’ (FGD 3)*.

#### Increased need for health care during emergencies

Participants indicated that the increased volume of patients at health centers limits the amount of time that can be spent treating each patient. Staff reported having to select the most urgent cases to treat and this decision is generally related to the level of physical injury. “*After a hurricane or a disaster, you might have a large amount of people coming here, let’s say you would stay 15, 20 minutes with somebody it’s going to be a rush on that day. So, you might spend about 2 minutes, 3 minutes trying to deal with each person, …. everything is going to be limited….’’ (FGD 2).* Participants suggested that the increase in workload that typically follows a climate related event, generally disrupts the normal functioning of the health services and limits access to care for many vulnerable groups. As a result, some people face challenges with medication stock-outs and inconsistencies in follow-up clinics.

#### Occasional limited access to medical supplies and health centers

Limited medical supplies at health facilities is also a barrier towards accessing health services. Participants indicated that there are generally insufficient supplies to allow enable community health centers to function adequately after a climate-related event. In many cases, medicines and other resources are stretched beyond their limits affecting the availability of services for many in the population and challenges such as medication stock outs are common. Staff noted that the resources provided to the PHC were generally so limited that patients with chronic diseases could not receive adequate supplies due to limitations of the PHC pharmacies. One participant noted “*I think access to medication was another problem, […] some of the persons in the village that didn’t have a stock up on medication [prior to the storm, and therefore] didn’t have medication for that month.” (FGD 5)*.

Some participants noted that some individuals have limited access to some health care facilities due to the location of facility. Some felt that not enough attention was being paid to ensuring accessibility for older people.

#### Focus on the biomedical and less on mental health and other determinants of health

Based on the discussions, it was understood that PHC practices mainly follow a biomedical approach to health care, focusing on the management of physical health problems. This is a cross-cutting theme related to the (lack of) assessment of risks and vulnerabilities. Participants noted that after an extreme climate event, the priority is to treat physical injuries such as broken bones, acute wounds and head injuries as well as acute cases of communicable diseases. Therefore, routine clinics for treatment of chronic diseases are generally rescheduled. It was clear that mental health problems are considered less important than physical injuries and, therefore receive less attention from health workers. When asked how care is prioritized in the PHC, one participant replied: *“You do your assessment and then you decide. Most times on the physical aspects you would see it. So, I’m saying yes somebody might be depressed but [on the other hand] somebody come with - let’s say a broken bone, you have to decide …. After all, it is quite obvious…” (FGD 2*). The example indicates that a broken bone would automatically take precedence over a mental health problem such as depression.

## Resource allocation

Participants described severe resource constraints within PHC that hindered their ability to provide quality health care services. Limitations in human resources for health, financial resources, and physical equipment pose challenges to the type and quality of services that the PHC is able to provide and these challenges are said to be chronic in the PHC.

In all the FGDs, participants lamented over the lack of supplies and limited human resources for health. This general perception of lack of resources seemed to be exacerbated during extreme climate events. Participants perceived that they are expected to provide efficient and good quality health services with limited physical and human resources and this situation has not changed despite the previous experiences with climate extremes. Resources are insufficient for the normal day-to-day functioning of the primary health care system: “*The resources were not there at the time to deal with the patient” (FGD 7).* Some participants felt that even the effort and time required to provide the necessary level of physical and psychological support was often beyond the capacity of staff. They expressed feelings of burnout and stress as a result.

Resource allocation was linked to access to care. Staff reported being overwhelmed, especially after extreme climatic events, due to the large influx of patients seeking care for acute problems: *“if access to one health center is compromised, the population served by that health center have (or need) to be redirected (to another facility) causing overwhelm at other health centers” (FGD 4)*. In addition, staff shortages meant that staff worked long hours with limited breaks and little relief. “*We can always offer the services but staffing is one of our major issues. If we do not have sufficient staff there is going to be a burden or the services will be less because is one nurse to (serve) one hundred or one nurse to 600 people in a community. The one-on-one care is still an issue because of staffing (FGD6)*.

Staff did not feel adequately supported during past climate disasters. The sense of being overwhelmed was exacerbated by the personal stressors faced by the staff and the feeling of being alone: “*In the district there was not enough backup to help. I was severely impacted personally and there was no backup for me from no area that I could take a day off so I could take care of my personal business because I had to come in tears and still had to operate.[…] That was hard” (FGD 3)*.

Working conditions for primary health care staff in the field, especially during climate extremes, were not ideal. Staff reported having to improvise in order to provide services, which increased their level of psychological stress. They cited several instances of working without the necessary resources: *“During Hurricane Maria I remember we had to use the counter to do dressings, there was no water (FGD1); “Without electricity, many people were still able to have their pap smears. You use a flashlight; you use your phone but we could still do pap smears” (FGD5)*. Participants perceived that the difficulties faced by staff both personally and in the execution of their work, precipitated an increase in migration of health workers. They felt that staff were treated poorly by authorities after the last two climate extremes and staff who could no longer endure the hardship left the country: *Disaster and climate change have affected the migration of our nurses because of the way the system treated us …. some persons they reached a peak.” (FGD 3).* The country then has to rely on foreign nurses from Cuba, which poses another challenge in terms of language barriers and effective communication. Some participants also emphasized the need for specialized training to enable PHC staff to function effectively. They explained that staff are transferred from the secondary care without adequate orientation to PHC, which affects their ability to perform the required tasks specific to PHC.

### Policy development

Participants were not aware of any policies within the primary health care system related to climate change. Staff are guided by the primary health care directives (2011) and the primary healthcare manual (1982). Each health district is required to develop a ‘disaster plan’ to guide staff response in the event of a hazard (e.g. a hurricane). Staff are engaged in the development of the disaster plan, but questions can be raised about its focus and systematic operationalization.

#### The limited use of monitoring and surveillance data for policy purposes

The previous themes of surveillance and monitoring and access to health care show that vulnerability is seen as consisting of demographic, social and medical components, and that the related information will somehow guide local field practice. However, they are not systematically treated at a central level as important knowledge for planning and practice. These potential geographic differences could lead to issues of inequity. Regarding vulnerability, some participants raised the issue of the lack of proper land use planning, which can potentially expose people and territories to climate hazards. This includes issues of communication and collaboration for cross-sectoral action to prevent or reduce vulnerability to climate events.

#### Limited consideration for PHC participation at the central level

Participants felt that there is a need for policy to address climate change issues and that this needs to take into account the advice of the staff: *“Our policy holders need to take our advice, because advice are given, they don’t listen and we find ourselves in problem (trouble)” (FGD 4)*. Participants expressed their dissatisfaction with the level of input that they have in policy and infrastructure matters. They cited examples of facilities which were being built with little input from PHC staff. One participant stated: *I think the policy makers should, when they (are) making new facilities they should involve their staff because it’s the staff that knows what essential part that they use in the facilities. For instance, we are there right now and that health center is (newly) built and there is no area that we can do dressings for persons and that health center was recently built (FGD 6).*

Some participants were concerned that PHC was not a priority in Dominica. *“I think they should give primary health care a little more priority, …we are there serving the public and there are no special preparations made, and you have no priority. There are no special vehicles to come pick you up and we are not getting no security. We should get more priority because we are serving the public” (FGD 5)*.

## Discussion

This study sought to better understand Dominica’s primary health staff’s perspectives on the adaptation of the primary health care system to climate change. Our study was inspired by the EPHF framework to guide data collection and organize results, according to its four core functions: assessment, access, resource allocation, and policy development related to climate change. PAHO’s newly adapted EEPHF framework includes environmental components that incorporate climate change and other environmental determinants.

Regarding assessment, this study confirmed what others have emphasized: there is an urgent need in the Caribbean for local data on climate change and other environmental determinants, and their relationship to health outcomes [9, 20]. In terms of monitoring and surveillance of climate-health issues, there is a need for greater collaboration with the meteorological unit to provide up-to-date weather and climate data (e.g. temperatures, precipitation) that could be used for public health analysis and planning. However, these data are not currently used to support the health assessment function, and the lack of systematic intersectoral collaboration impede the necessary adaptation to CC.

Based on the EEPHF framework, the health sector should include monitoring and surveillance activities on climate extremes, temperatures, water availability and quality, climate sensitive diseases, as well as the inclusion of socioeconomic factors and health outcomes. The current focus on hurricanes does not incorporate other potential health and social impacts of climate change such as sea level rise, declining river water levels, and heat waves into health planning and policy. Response and preparedness activities in the PHC system are limited to ‘hurricane response’ which, while important, may limit the ability of the system to adopt strategies and policies that lead to a more preventive and transformative orientation [36].

These findings underscore what other authors and reports have already and importantly recommended: the need for intersectoral collaboration and coordination among actors and sectors in the Caribbean [18], including in the primary health care system [37], as a key stakeholder in the process for climate change adaptation [1, 38]. Through their observations health care providers suggest that climate change is affecting the health of the population. These include infectious and vector-borne diseases, heat and respiratory problems, food security, and mental health, issues that have already been identified in other settings [3, 39] and in a recent study in Dominica.

Consistent with the findings of Macpherson (2013), the participants noted that the distinction between the wet and dry seasons was disappearing [40]. This was seen as having potential implications for food security on the island. This link was made in some FGDs where participants were concerned that the potential decline in agricultural production associated with climate change could contribute to an increase in the burden of NCDs on the island. Importantly, participants noted that there is currently no epidemiological data to confirm the link between climate change and NCDs. This is an important issue that needs to be addressed in order to better understand the impact of climate change on public health and to guide the development of policies that support adaptation to climate change in Dominica and more broadly across the Caribbean islands. It points to the need for improved surveillance including socio-environmental indicators, and research on the links between climate events and health outcomes to guide the development of policies and programs for adaptation to climate change in Caribbean SIDS [37]. The need to improve the health information system was identified by PAHO several decades ago, not only in Dominica but also in other English-speaking Caribbean countries. The lack of data on hazards and threats, inadequate use and analysis of local health data, lack of relevant indicators to monitor issues related to quality of care and morbidity, and lack of feedback for activity planning and policy are all issues that have been highlighted in multiple studies and reports in Caribbean SIDS [18].

Access to care is an important issue in Dominica’s PHC system. An assessment of the PHC following Hurricane Maria in 2018 reported that services within the primary health care system were fragmented and incomprehensive due to a focus on curative care and that some staff perceived PHC services as “generally ineffective” particularly with regard to noncommunicable diseases [41]. This supports our interpretation of the lack of emphasis on health promotion. PHC services are disrupted during climate extremes and as seen during Tropical Storm Erica and Hurricane Maria, these disruptions can last for several months, affecting the population’s access to services [42]. Additionally, individuals, including pregnant women, reported having to seek healthcare outside of Dominica after Hurricane Maria due to disruptions in health care services [16]. Furthermore, in their assessment of the PHC in Dominica using the Primary Care Assessment Tools (PCAT), Macinko et al. postulated that health care providers may lack knowledge about populations not accessing services, as there was a large disparity between the access to care scores as rated by user’s and healthcare providers. Moreover, user ratings of access to care were much lower than what is perceived by the health care providers [43]. The building of smart health centers on island is potentially interesting given the focus on access to services and climate resilient buildings/infrastructure, and warrants further investigation [21].

The assessment of the social determinants of health, which is seen as a public health function to ensure equity access to health care, were rarely raised and not really discussed in the FGDs. Many people displaced in Dominica after Hurricane Maria [11] could have experienced situations that exacerbate or create precarious living conditions [16]. The Kalinago people – the indigenous people of Dominica – have not been mentioned in discussions of vulnerability to climate change. However, the poor living conditions that affect the Kalinago territory [44] are likely to make them vulnerable to climate change. Thus, the perspective that vulnerability to climate extremes is politically and socially produced [6, 45] does not seem to be considered. The most common categories identified by primary health care professionals as vulnerable are rather static and include the children under 2 years of age, pregnant women, shut ins (home bound people), and patients with chronic diseases. However, it is clear that policies and programs (or lack thereof) have the power to influence the social vulnerability of populations to climate extremes and affect health. This raises the issue that some circumstances may lead to families and/or communities becoming increasingly vulnerable according to their exposure and sensitivity to the climate event and, consequently, producing social inequalities in health. This is an important issue to understand, as public health policies and programs can potentially reduce vulnerability to climate change by increasing the adaptive capacity of communities [45]. A lack of focus and data on vulnerability and equity in access to quality health care [18] and social inequalities in health more generally [46] have already been identified as key concerns for the English-speaking Caribbean.

The issue of mental health is not addressed in the EEPHF. However, authors recommend the development of a mental health program in Dominica, as an extreme event such as a storm or a hurricane can potentially create insecurities and uncertainties regarding housing, livelihoods, and water and food availability which can potentially affect mental health [16]. The need for community mental health services along with other health care services has already been identified in Dominica [18]. These findings therefore call for health professionals to take a holistic view of health and to act accordingly in terms of disease prevention and health promotion, epidemiological surveillance, and ensuring the most inclusive possible access to health care, with a particular focus on the environmental and social determinants of vulnerability [16]. Participants in the study reiterated that there is an extreme shortage of human resources for health, infrastructure deficiencies, and constraints on other resources for carrying out daily healthcare activities, which inhibit the ability to carry out basic operations of PHC facilities. It has already been noted that in the Caribbean region, there is a long-term problem of lack of equipment and shortages in the number and quantity of different categories of personnel, as well as the poor distribution of personnel within the health care system [18, 47]. The importance of the health workforce has been highlighted in building capacity for climate resilience [48]. The consequence of not addressing long-term problems is that they are exacerbated during periods of instability, as was seen during the COVID-19 pandemic [47].

In general, none of the PHC staff could identify any climate-related policies that have been developed or implemented in Dominica. A Climate Resilience Plan has been developed for the Government of Dominica with support from the World Bank [34]. However, there is no section on public health and the health system in Dominica. Social participation and the mobilization of civil society and communities to promote communication and action are considered as important for the policy development phase of the EEPHF [21]. There is also evidence that public health in the Caribbean should be more proactive, provide leadership on climate change and health, and be involved in policy development about climate change [49]. There is also strong evidence that health professionals are key stakeholders who should be involved in health policy development [50]. Adaptation should be based on both scientific and local knowledge [31]. Primary health care centers are well positioned to build relationships and collaborate with communities to understand and analyze the links between the broader environment and population health. Communities have valuable insights into their living context [51], as local and/or traditional beliefs may inhibit adaptive capacity [52]. However, the question arises as to the political conditions that can ensure such engagement and commitment of all social and health actors, including communities in their contribution to climate change adaptation. In the Caribbean, a lack of community engagement and participation in health issues has already been identified [18]. We believe that it is important that the knowledge and potentially beneficial practices that may result are made possible by the creation of an enabling environment. At present, it is not certain that these conditions exist in Dominica [16], and it would be appropriate to investigate this issue. In addition, there is a need for capacity building in the public health sector, including education and training of public health professionals on the health risks of climate change [9]. Finally, there is evidence that adaptation to climate change needs to include policies and multiple resources directed at the primary health care system, which could lead to an improvement in the adaptive capacity of communities [37]. However, some authors suggest that, in some settings, policymakers underestimate the important role of primary health care in promoting and protecting the health of the population. Health authorities in the Caribbean region should advocate for the strengthening of primary health care systems [53]. Similarly, we believe it is important to improve major organizational problems related to the health system, that have already been identified in the English-speaking Caribbean: these include centralization of power within the health system, feedback that follows professional lines but is not integrated to all health care workers (fragmentation), and, in health districts, responsibility of staff without the necessary authority [18].

This study has several strengths and limitations. To our knowledge, this is the first qualitative research on primary health care and climate change conducted in Caribbean SIDS. It provides valuable information and important feedback in the process of assessing adaptation to climate change, especially in the domain of public health and primary health care. One FGD had only 5 participants, although the majority had at least 7. Although we encouraged everyone to participate, it is possible that the critical perspectives of some participants were not expressed for various reasons. This study did not have the opportunity to investigate specific health outcomes. Taking into consideration both the peculiarities and heterogeneity of Caribbean SIDS, and more generally SIDS, some of the recommendations that pertain to Dominica, may be applicable in other and similar SIDS settings.

## Conclusion

This study suggests important concerns regarding the primary health care system in Dominica that relate to essential public health functions: assessment, access, resource allocation, and health policy. Primary health care is an important stakeholder in climate change adaptation, but it is not receiving the attention that it deserves from decision makers. Our study suggests numerous gaps in the activities and implementation of the primary health care in Dominica, most of which have been previously raised a several decades ago for the English-speaking Caribbean [18]. Climate change and related events have the potential to severely constrain the PHC system, exacerbating barriers to access to health services and deepening social inequalities in health. Implementation of the EEPHF framework requires institutional support, and interdisciplinary, and inter sectoral collaboration [21]. Climate change adaptation requires political commitment, clear institutional frameworks and policies, and adequate funding [1], as well as overall institutional capacity building [9] as essential prerequisites for adaptation to climate change with the support of high-income countries [54]. In the Caribbean, there is a need to strengthen public health leadership [44], especially in climate action [49]. Therefore, public health should be a key actor to contribute to the design and operationalization of adaptation and transformative resilience to climate change. It has already been noted that in the Caribbean, there is a need for policies and practices that incorporate the reduction of social inequalities, and promote equity and social justice as core values of public health [46]. In primary health care, the biomedical perspective of health should be broadened to a public health approach that includes the linkages between environmental and the social determinants of health [3, 21]. Strengthening primary health care could help make communities more resilient to climate change [37]. There is an urgent need to pay more attention to the use of ecosystems and biodiversity which pose a threat to water and food security, and human health [54]. Finally, future research could explore specific strategies for climate action and specific diseases, community engagement and participation, ways to build public health capacity, strategies to build leadership in public health and primary health care, relevant epidemiological indicators for climate and health surveillance and monitoring, ways to improve access to health care, tools to measure vulnerability to climate change in primary health care, and policies needed for climate change adaptation.

### Electronic supplementary material

Below is the link to the electronic supplementary material.

Supplementary Material 1

## Data Availability

The dataset for this study is not publicly available due to stipulations of ethical approval but can be made available from the corresponding author upon reasonable request.
