# Supplementary material for: “I think they should give primary health care a little more priority”. The primary health care in Caribbean SIDS: what can be said about adaptation to the changing climate? The case of Dominica— a qualitative study
Source: BMC Prim Care. 2024 Feb 22;25:65. doi: 10.1186/s12875-024-02311-w (PMC10882746; doi:10.1186/s12875-024-02311-w)
Supplement: Supplementary file 1 — Supplementary Material 1 [file 12875_2024_2311_MOESM1_ESM.docx]

Interview guide with PHC staff, Dominica (*CliMiHealth*)

**INTRODUCTION**

We are interested in hearing your perspective on issues related to climate change, the primary health care system and the community to which you provide care. More specifically we are interested in your view on climate change, how it affects the PHC and the population. Our study aims at contributing to the adaptation to climate change in the West Indies.

**PERCEPTION OF CLIMATE CHANGE**

1. *What would you say about climate change in Dominica?*
2. *How do you think it affects the health of population?*

**PREPAREDNESS, RESPONSE, REORGANIZATION**

1. *If you think of TS Erika (2015) and Hurricane Maria (2017) what are the differences in interventions?*

***Probe:*** *what are the lessons learned based on the interventions used? What are the strengths and weaknesses in interventions?*

**HEALTH ASSESSMENT**

If you think of TS Erika (2015) and Hurricane Maria (2017)

1. *How were you able to identify the individuals in need of health services in your community?*
2. *How did you cope with health risks/threats in your community?*

**PROVISION OF HEALTH CARE SERVICES**

If you think about Erika (2015) and Maria (2017), *did you identify*:

1. *How these events have impacted the functioning of primary health care ?*

Probe: How difficult was it *to provide* ***health care*** *to the community?* e.g., lack of physical and human resources

1. *Were there specific barriers for people to access health care?* [e.g. transportation, landslides, etc.]
2. *What about people with specific vulnerabilities*?

**Probe:** Including those who have been displaced?

**POLICY DEVELOPMENT**

In relation to climate change and climate events adaptation for health system (storms, heat waves or floods, etc)

1. *Has anything been formulated by a health authority regarding climate change and PHC*? (support, materials, training, resources, collaboration, etc.)

Probe:

-To prepare and respond to climate events such as storms, heat waves or floods

-To assign e.g. responsibilities/roles of workers (alert phases, impact, evacuation, triage, relocation, support in the first hours or weeks)

If yes: Can you explain some of the proposed strategies to ?

If no: Are there any strategies proposed by Ministry of Health? What are some of the points that stand out to you?

1. *Do you ensure partnerships with communities or any other stakeholders in relation to climate change adaptation?*

-How? To do what? Was it positive/negative?

**TRAINING RECEIVED AND QUALIFICATIONS REQUIRED** [PREPARATION, COMPETENT WORKFORCE]

1. *Have you ever attended any training regarding preparedness and response to extreme climate events* (like storms or hurricane)?

**Probe:** *If yes, can you tell me about the training that you received?*

*If no, what kind of training would you need in this domain*?

**RECOMMENDATIONS [potential transformation]**

1. *What recommendations would you like to make to various stakeholders (community, district, ministry, government, etc.) regarding PHC and climate change in the future*?
2. *Do you have anything to add*?
